# How Significant Are Xpert Xpress SARS-CoV-2 Test Findings When Only an N2 Gene Is Detected?

**DOI:** 10.3390/diagnostics12092133

**Published:** 2022-09-02

**Authors:** Min-Kyung So, Hae-Sun Chung, Duk Hee Lee, Miae Lee

**Affiliations:** 1 Department of Laboratory Medicine, Ewha Womans University College of Medicine, Seoul 07985, Korea; 2 Department of Emergency Medicine, Ewha Womans University College of Medicine, Seoul 07985, Korea

**Keywords:** COVID-19, emergency medicine, rapid screening, qRT-PCR, SARS-CoV-2, Xpert

## Abstract

The rapid identification of patients infected with COVID-19 during the SARS-CoV-2 pandemic is critical to operating emergency rooms effectively. Xpert Xpress SARS-CoV-2 (Xpert) assays are increasingly being used in the rapid screening of COVID-19. We evaluated the clinical performance of Xpert by comparing findings with those of qRT-PCR evaluations and included the clinical features of patients visiting the emergency department. Positive results with Xpert testing (n = 370) were compared with qRT-PCR findings, demonstrating a 91.9% intertest agreement. We reviewed the subsequent COVID-19 test results and SARS-CoV-2 infection histories for individuals showing discrepancies in Xpert and qRT-PCR testing and determined whether the findings were true-positive or false-positive. The true-positive rate for Xpert testing was 95.4% (353/370); the remaining 17 samples (4.6%) were false-positive. All false-positive data for Xpert testing showed N2 signals amplified to Ct values of ≥40 with no E gene signals. Rapid Xpert testing is highly sensitive and shows a good performance overall in challenging situations, such as an emergency room. However, we considered the possibility of false-positive Xpert results given an N2 gene signal only, especially given high Ct values. We recommend interpreting test data with caution and considering retesting over time.

## 1. Introduction

The severe acute respiratory syndrome coronavirus 2 (SARS-CoV-2) pandemic has increased the need for rapid molecular testing in emergency departments (EDs) in order to provide timely results for the purpose of rapid therapeutic intervention [1,2,3]. 

The Xpert Xpress SARS-CoV-2 (Xpert) assay is an automated molecular test based on nucleic acid amplification that integrates the specimen processing and target amplification steps. This test can provide a rapid result within one hour [4]. The Xpert assay is often selected for SARS-CoV-2 RNA detection in situations when timely infection control and rapid therapeutic intervention are required [5].

This test has been shown to not only shorten the turnaround time for obtaining SARS-CoV-2 testing results, but also to provide improved sensitivity compared to antigen tests and other rapid detection tests with regard to the detection of the SARS-CoV-2 virus [4,6,7,8]. 

The Xpert assay targets the nucleocapsid (N2 region of the N gene) and envelope (E) genes of SARS-CoV-2, providing positive results when the cycle threshold (Ct) value is less than 45 cycles for both nucleic acid targets (N2 and E) or when the Ct value for N2 alone is below 45. Xpert results are interpreted automatically according to the manufacturer’s instructions. The Xpert Ct threshold is a delayed one from laboratory-based real-time reverse transcription-polymerase chain reaction (qRT-PCR) tests (which present with typical Ct cut-off values of 35–40) [9]. 

There have been notable reports of cases testing positive with only the N2 region observed in samples demonstrating a very low viral RNA load; this has raised the possibility of false-positive results [10]. However, comparing results across different platforms could be insufficient to address this question. At our institution, several patients demonstrated positive results following analysis via the Xpert assay for which only the N2 gene was amplified, yet there was no traceable evidence of coronavirus disease 2019 (COVID-19) infection in some of the cases.

Given the above findings, this study aims to evaluate the clinical significance of Xpert testing results by comparing findings for Xpert and qRT-PCR (an approved and standard testing modality) overall and in reference to clinical information in Xpert-positive individuals, especially in those for whom Xpert testing detects only the N2 gene.

## 2. Materials and Methods

### 2.1. Study Population and Data Collection

Among 11,737 patients, we selected 566 that demonstrated positive results following analysis via the Xpert SARS-CoV-2 assay (Cepheid, Sunnyvale, CA, USA). Sample collection was performed using a nasopharynx swab in patients visiting the ED at our hospital between May 2021 and April 2022. Among the 566 Xpert-positive patients, 333 with a positive E and N2 detection result and a comparable qRT-PCR result obtained within 24 h (mean ± standard deviation: 2.38 ± 3.92 h) and 71 with a positive N2-only detection result were selected for study inclusion. The 34 patients (17 with a positive E and N2 detection result, 17 with a positive N2-only detection result) that could not be retested because of a lack of remnant samples were excluded from the present study. Finally, a total of 370 SARS-CoV-2-positive patients with positivity determined according to findings obtained using the Xpert assay were included in the present data analysis, as described below. 

### 2.2. Threshold for Positivity and Interpretation

Per manufacturer instructions, Xpert test results were interpreted as positive if the E and N2 genes, or the N2 gene alone, demonstrated Ct cut-off values ≤ 45. This test provides a presumptive positive result when only the E gene is detected, owing to the similarity of this target to other coronaviruses belonging to the Sarbecovirus subgenus of the Coronaviridae family [11]. 

We used two kits to conduct qRT-PCR testing: (1) an Allplex SARS-CoV-2 (Allplex) assay (Seegene, Seoul, South Korea) targeting the Sarbecovirus E gene, the SARS-CoV-2 RNA-dependent RNA polymerase (RdRp) gene, the S gene encoding spike protein, and the N2 gene; and (2) a Standard M nCoV Real-Time Detection kit (SD Biosensors, Suwon, Korea) targeting two regions in the E and ORF1ab (RdRp) genes. The qRT-PCR methods were performed according to manufacturers’ instructions. Assay results were considered positive if all targets were amplified with Ct values ≤ 40 for the Allplex assay and ≤36 for the Standard M assay. The sample was considered “inconclusive” if only one or two gene targets were amplified with Ct values ≤ 40 for the Allplex assay or one gene target was amplified with a Ct value ≤ 36 for the Standard M assay. 

### 2.3. Evaluating Agreement and Clinical Performance

We evaluated intertest agreement between the Xpert and qRT-PCR assays using tests performed within 24 h (mean ± standard deviation: 2.38 ± 3.92 h). If the results between the Xpert and qRT-PCR assays were discrepant, such as results with Xpert-positive–qRT-PCR-negative or Xpert-positive–qRT-PCR-inconclusive, or if the Xpert-positive results showed an N2-only signal, we retested the sample using a qRT-PCR Allplex assay. In order to exclude the possibility of time effects or differences in sample quality, we readministered the Allplex assay using original specimens that were stored frozen at −70 °C (i.e., the specimens obtained for Xpert testing). 

Among the discrepant results, we excluded data for samples that could not be retested owing to a lack of sample availability from further analysis. For calculating concordance, we considered an inconclusive or positive result from qRT-PCR testing as indicating agreement with the positive result found with Xpert testing. 

For evaluating clinical performance, we reviewed the results obtained from COVID-19 testing using Xpert or qRT-PCR assays conducted using a nasopharyngeal swab or sputum samples performed within one month after visiting the ED. We defined true-positive Xpert findings as confirmed in patients with a history of SARS-CoV-2 infection within 90 days prior to their ED visit or according to the presence of positive or inconclusive results on at least one COVID-19 test using a qRT-PCR assay conducted within two weeks following Xpert testing. 

According to existing reports, SARS-CoV-2 RNA can be detected in upper respiratory tract samples for up to 90 days after symptom onset [12]. Based on these studies, we set the cut-off for the number days elapsing until diagnostic confirmation for preconfirmed cases to within 90 days. Since the incubation period for coronavirus is between two and 14 days, if the virus was detected on COVID-19 testing conducted within two weeks after Xpert testing, we considered that the same virus was detected [13]. If there was no COVID-19 history and all reviewed qRT-PCR results were likewise negative, the finding was considered an Xpert false-positive finding. 

Moreover, we reviewed each individual’s symptom presentation on further clinical evaluation. Symptoms and signs included fever, cough, sputum production, sore throat, peripheral oxygen saturation ≤ 93%, headache, and general weakness. 

Statistical analyses were performed using MedCalc Statistical Software version 20.013 (MedCalc Software Ltd., Os-tend, Belgium).

## 3. Results

### 3.1. Concordance of the Xpert Test with the Standard qRT-PCR Test 

A total of 370 SARS-CoV-2-positive patients tested using Xpert assays were evaluated in the present study. Two targets were detected and reported as positive in 316 of these patients. The remaining 54 patients showed positive results with only an N2 signal. The Ct values for N2 ranged from 37.7 to 44.4. There were no presumptive positive results showing only an E gene signal. By comparing data collected using both assays, Xpert-positive samples (i.e., E and N2) were positive on confirmation with qRT-PCR testing in 305 (96.5%) patients and were inconclusive in 11 (3.5%) patients (Table 1). 

For the 11 patients with inconclusive qRT-PCR results, the mean Ct value for the E gene was 38.2 ± 3.7 on Xpert testing, while that for the N2 gene was 38.8 ± 2.6. When both E and N2 targets were detected on Xpert testing, there were no qRT-PCR-negative results. However, in qRT-PCR assays of the 54 samples in which Xpert testing detected only the N2 gene, the findings were positive on confirmation (i.e., demonstrating concordance) in 9 (16.7%) patients and were inconclusive (also considered as demonstrating concordance) in 15 (27.8%) patients. The remaining 30 samples yielded discordant results, showing negative results on qRT-PCR testing (55.5%). We calculated the overall intertest agreement as 91.9% (95% CI, 88.7% to 94.3%), as shown in Table 1.

### 3.2. Evaluating True-Positive and False-Positive Xpert Test Results 

To further focus on Xpert data presenting with an N2 signal only, we reviewed each patient’s follow-up COVID-19 test results and clinical history. The detailed information and interpretation of the 54 samples with only N2-gene-positive results is provided in Table S1 (Supplementary Materials). Of these 54 samples, 37 (68.5%) were categorized as true-positive findings. The remaining 17 samples (i.e., 31.5% of the N2-only positive samples) were categorized as false-positive findings (Table 2). 

Among the 30 discordant samples (i.e., the samples that were Xpert-positive with only the N2 target detected but were qRT-PCR-negative), 13 were determined to be Xpert true-positive findings. In 3 out of these 13 samples, we initially obtained qRT-PCR-negative results but later obtained inconclusive or positive results. Therefore, we considered that Xpert testing yielded early detection. Moreover, of the 13 samples, 10 samples were determined to be preconfirmed cases of SARS-CoV-2 infection within the past 90 days on reviewing patient medical records. Therefore, these patients were also categorized as showing Xpert true-positive findings (Table S1). Overall, 95.4% (95% CI, 92.8% to 97.1%) of the Xpert-positive samples were categorized as Xpert true-positive findings.

We investigated Xpert findings with only N2 gene detection according to patients’ previous history of SARS-CoV-2 infection or lack thereof and compared these values between symptomatic and asymptomatic individuals (Table 3). The percentages of symptomatic individuals were statistically significantly different between Xpert false-positive (11.8%, 2/17) and true-positive samples (40.5%, 15/37) (*p* = 0.0366). Most false-positive samples in individuals with no SARS-CoV-2 infection history were derived from asymptomatic cases. However, the association between symptomology and qRT-PCR findings did not demonstrate any differences according to SARS-CoV-2 infection history (*p* = 0.6428). 

**Figure 1:**
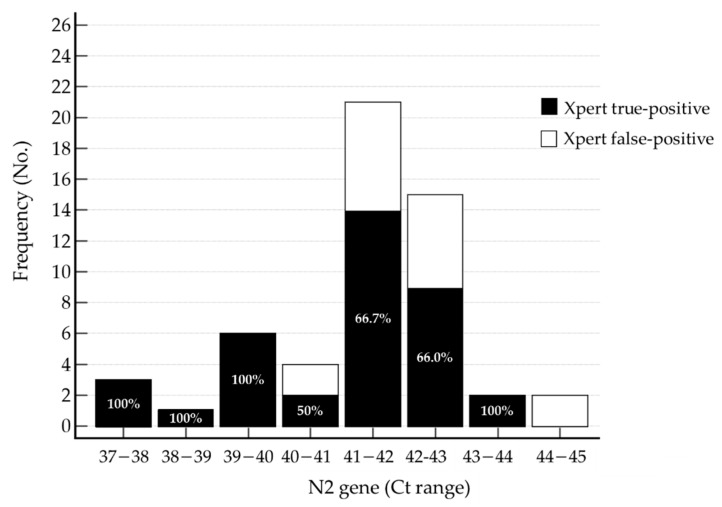
Xpert true-positive percentages according to the N2 gene Ct range (within a valid range, Ct value < 45) in E-gene-negative and N2-gene-positive samples. Abbreviations: Ct, cycle threshold; E, envelope gene; N2, nucleocapsid gene 2; SARS-CoV-2, severe acute respiratory syndrome coronavirus 2; Xpert, Xpert Xpress SARS-CoV-2 assay.

We were interested in the cut-off Ct values in true-positive samples for Xpert findings detecting only the N2 gene. After categorizing according to Ct values for the N2 gene in the positive samples detecting only the N2 gene, false-positive samples were found among those with N2 Ct values ≥40. When the N2 Ct value was <40, 100% of the Xpert results were true-positive findings (Figure 1). There were statistically significant differences when comparing the true-positive proportions of the two groups using the cut-off of a 40 Ct value (*p* = 0.0187).

## 4. Discussion

In the present study, we provided concise, summative information on Xpert-positive data from patients presenting at our ED in comparison to standard qRT-PCR test findings and follow-up COVID-19 tests. When comparing Xpert data to qRT-PCR data, the overall positive percentage of agreement was 91.4%. By combining the COVID-19 follow-up test results and information on patient history of SARS-CoV-2 infection, the true-positive rate for Xpert testing increased to 95.7%. Our findings are similar to those reported in the previous literature, wherein agreement with the Allplex 2019-nCoV assay was determined at 95.6% [14]. 

Xpert tests are performed in the emergency room if a patient is suspected of having COVID-19-related symptoms, if a possibility of re-infection after release from quarantine due to SARS-CoV-2 infection is suspected [12], or if a patient needs preemptive COVID-19 testing for COVID-19-unrelated rapid diagnosis and treatment. The positive rate of the Xpert tests performed in our study was 4.8% (566/11,737). In approximately 10% of COVID-19-positive samples, we detected only an N2 signal. Khoshchehreh et al. reported that only 3.9% of Xpert-positive findings showed only an N2 signal from among the total positive test findings [15]. In contrast, Falasca et al. reported that 38.8% of Xpert-positive findings showed only an N2 signal from among the total Xpert-positive findings presented in their study [10]. The prevalence and properties of Xpert-positive findings showing only an N2 signal can vary across studies and settings according to the testing environment and the available participant pool. For example, although the Xpert assay is designed to minimize the likelihood of a false-positive test result, this test could give a false-positive result when used in locations with less than a 5% prevalence [16]. 

Discordant results can occur for various reasons, including differences in sample collection, the timing of testing after exposure to the virus, and differences in assay sensitivity [17,18,19,20]. The experiences of several research teams have shown that sampling procedures contribute significantly to false-negative results on COVID-19 tests. In addition, inconsistent results have been found in patients evaluated at various timepoints of infection who have the commonality of a low SARS-CoV-2 viral load [18,19]. In this study, residual samples from the Xpert test were used when retesting discordant samples with qRT-PCR. We thereby reduced concerns regarding the potential poor quality of specimens or differences in the timing of specimen collection. 

The Xpert assay detects the pan-Sarbecovirus E gene, as well as the N2 region of the N gene. In our comparisons between Xpert findings and comparative standard qRT-PCR findings, the 316 Xpert-positive samples detecting both the E and N2 genes showed 100% agreement with the qRT-PCR results showing positive or inconclusive findings, demonstrating a high possibility of the presence of SARS-CoV-2 RNA given these positive Xpert test results. 

However, 55.5% (30/54) of the Xpert-positive samples detecting only an N2 signal showed negative results for qRT-PCR testing using the same specimen. We were interested in the discrepancy data. On further investigation, 13 out of 30 cases were demonstrated to be Xpert true-positive findings. Of these, 10 were preconfirmed cases, namely, these were cases presenting with a history of SARS-CoV-2 infection within the past 90 days. Consequently, it was thought that low amounts of RNA remained in these patients. We did not detect any SARS-CoV-2 RNA on qRT-PCR testing in eight of these cases. Therefore, we concluded that positive findings were detected only in regard to the N2 gene on Xpert testing, detecting this finding with high sensitivity. 

In contrast, in the other three cases, Xpert testing could detect the presence of SARS-CoV-2 RNA earlier than when employing qRT-PCR. All three patients visited the ED without a history of SARS-CoV-2 infection and had no COVID-19-related symptoms. Two to four hours later, COVID-19 results from these patients detected at least one target signal on qRT-PCR testing. Therefore, we could consider these to be early-detection cases through the Xpert assay in patients who truly had COVID-19 infections [21]. The possibility of the early detection of SARS-CoV-2 using the Xpert platform could be of great interest, including in the ED environment, because this methodology could allow for a prompt response in regard to diagnosis and effective and timely treatment in critical cases [3].

We noted that 17 out of 30 patients had no COVID-19 infection history and no further COVID-19-positive results and were, therefore, considered to have Xpert false-positive findings according to our aforementioned criteria. The findings of previous reports are also in support of our data [14]. More specifically, these reports demonstrated the possibility of Xpert false-positive findings occurring when only an N2 signal was detected through the virus enrichment process in samples with high N2-associated Ct values on Xpert testing (Ct > 38). All cases (3/3) with both E and N2 signals were proved as true-positive findings. However, only 2 out of 14 samples showing an N2 signal only demonstrated true-positive findings. 

Most of our false-positive cases did not present with any COVID-19-related symptoms. We noted that false-positive findings occurred more often with high N2 Ct values over 40, which was probably related to nonspecific amplification. One research team previously suggested that the Ct values of the E and N2 targets should be set to 40 instead of the current manufacturer-specified value of 45 in order to exclude the possibility of false-positive findings given high Ct values [22]. We recommend that clinicians exercise caution when interpreting Xpert results with high N2-associated Ct values over 40 if the Ct value is provided. If COVID-19 infection is still suspected according to clinical symptomology, we recommend confirming the test findings using alternative methods or retesting.

This study had some limitations. First, when distinguishing true-positives from false-positives, additional COVID-19 test results were not available for some patients. Therefore, some of the data were classified only according to a comparison of results between Xpert and qRT-PCR assays conducted within 24 h. Second, even if qRT-PCR showed an inconclusive result, it was regarded as indicative of the presence of SARS-CoV-2 RNA, and the Xpert result was classified as a true-positive. However, we note that our data could not exclude the possibility of false-positive findings for qRT-PCR testing. 

## 5. Conclusions

Our results demonstrated that the rapid Xpert assay had the potential to detect very low viral RNA loads earlier and with higher sensitivity compared to routine laboratory qRT-PCR tests. Therefore, the assay could be useful in EDs, where rapid COVID-19 diagnosis and management are needed. However, this methodology also carried the possibility of false-positive findings. Hence, we recommend that clinicians exercise caution when interpreting Xpert test results with only N2 gene detection, especially those with high Ct values, and consider alternative methods or retesting. 

## Figures and Tables

**Table 1:** Comparative results for the Xpert and qRT-PCR assays conducted using nasopharyngeal swab clinical samples.

	% (No.) of Results on qRT-PCR		
	Positive	Inconclusive	Negative
Xpert-positive with only an N2 signal	16.7% (9/54)	27.8% (15/54)	55.5% (30/54)
Xpert-positive with both E and N2 signals	96.5% (305/316)	3.5% (11/316)	0.0% (0/316)
Xpert-positive, total	84.9% (314/370)	7.0% (26/370)	8.1% (30/370)

Abbreviations: E, envelope gene; N2, nucleocapsid gene 2; qRT-PCR, real-time reverse transcription-polymerase chain reaction assay; Xpert, Xpert Xpress SARS-CoV-2 assay.

**Table 2:** Clinical performance of the Xpert assay in SARS-CoV-2-positive individuals.

	% (No.) of True-Positive Findings *	% (No.) of False-Positive Findings
Xpert-positive with only an N2 signal	68.5% (37/54)	31.5% (17/54)
Xpert-positive with both E and N2 signals	100.0% (316/316)	0.0% (0/316)
Xpert-positive, total	95.4% (353/370)	4.6% (17/370)

Abbreviations: E, envelope gene; N2, nucleocapsid gene 2; qRT-PCR, real-time reverse transcription-polymerase chain reaction assay; SARS-CoV-2, severe acute respiratory syndrome coronavirus 2; Xpert, Xpert Xpress SARS-CoV-2 assay. * True-positive: individuals with results consistent with comparative qRT-PCR results, presenting with a history of SARS-CoV-2 infection within the past 90 days, or with positive or inconclusive results on at least one COVID-19 test conducted using a qRT-PCR assay within two weeks following Xpert testing.

**Table 3:** Percentage of symptomatic individuals with E-gene-negative and N2-gene-positive Xpert findings.

qRT-PCR Results within Two Weeks after Xpert Testing		No Previous SARS-CoV-2 Infection History	Preconfirmed *
At least one positive or inconclusive result	Total No. of individuals	11	18
	No. (%) of symptomatic individuals ^†^	2 (18.2%)	10 (55.6%)
No positive results	Total No. of individuals	17 ^‡^	8
	No. (%) of symptomatic individuals ^†^	2 (11.8%)	3 (37.5%)

* Within 90 days after an initial diagnosis of COVID-19. ^†^ Symptoms included fever, upper respiratory infection (URI) symptomology (cough, sputum, sore throat), oxygen desaturation, headache, and general weakness. ^‡^ The category was considered as Xpert false-positive, whereas the other categories were considered as Xpert true-positive. Abbreviations: E, envelope gene; N2, nucleocapsid gene 2; qRT-PCR, real-time reverse transcription-polymerase chain reaction assay; SARS-CoV-2, severe acute respiratory syndrome coronavirus 2; Xpert, Xpert Xpress SARS-CoV-2 assay.

## Data Availability

Data for this study, though not available in a public repository, can be made available upon reasonable request.

## Supplementary Materials

The following supporting information can be downloaded at: https://www.mdpi.com/article/10.3390/diagnostics12092133/s1, Table S1: Interpretation of results obtained from individuals with SARS-CoV-2-positive showing detection of only the N2 gene on Xpert testing.
