# Supplementary material for: How Significant Are Xpert Xpress SARS-CoV-2 Test Findings When Only an N2 Gene Is Detected?
Source: Diagnostics (Basel). 2022 Sep 2;12(9):2133. doi: 10.3390/diagnostics12092133 (PMC9498257; doi:10.3390/diagnostics12092133)
Supplement: Supplementary file 1 [file diagnostics-12-02133-s001.zip › diagnostics-1818448-supplementary.pdf]

Table S1. Interpretation of results obtained from individuals with SARS-CoV-2-positive showing detection of only the N2 gene on Xpert testing.

Individual No.	Xpert			Allplex assay				Clinical information and F/U COVID-19 data				Finial Interpretation of Xpert result
	E (Ct.)	N (Ct.)	Results	E (Ct.)	RdRp/S (Ct.)	N (Ct.)	Results	Initial or pre-confirmed diagnosis	Xpert testing after COVID-19 diagnosis	*F/U COVID-19 test	COVID-19-related symptoms & sign	
No.1	0	37.7	Positive	36.62	0	35.26	Inconclusive	Pre-confirmed	about 50 days	**I**	None	True positive
No.2	0	37.8	Positive	0	0	37.89	Inconclusive	Initial		**I**/N	None	True positive
No.3	0	37.9	Positive	34.17	36.17	33.88	Positive	Pre-confirmed	29 days	**P**/P/I/P/I/P/P	General weakness	True positive
No.4	0	38.9	Positive	38.10	37.66	38.97	Positive	Pre-confirmed	22 days	**P**	Pneumonia	True positive
No.5	0	39.1	Positive	0	38.06	0	Inconclusive	Initial		**I**/N	None	True positive
No.6	0	39.4	Positive	36.31	0	36.44	Inconclusive	Pre-confirmed	39 days	**I**/P/N/P/I	None	True positive
No.7	0	39.5	Positive	38.08	0	37.93	Inconclusive	Pre-confirmed	13 days	**I**	None	True positive
No.8	0	39.6	Positive	35.78	35.96	34.91	Positive	Initial		**P**/P	None	True positive
No.9	0	39.7	Positive	34.90	35.69	35.40	Positive	Pre-confirmed	26 days	**P**/I	None	True positive
No.10	0	39.9	Positive	34.09	35.81	34.60	Positive	Pre-confirmed	37 days	**P**/P/N(sp)/I	Sore throat	True positive
No.11	0	40.3	Positive	35.81	0	34.62	Inconclusive	Pre-confirmed	9 days	**I**/P/P/P/N	None	True positive
No.12	0	40.3	Positive	0	0	0	Negative	Initial		**N**	None	False positive
No.13	0	40.3	Positive	0	0	0	Negative	Pre-confirmed	15 days	**N**/I	URI symptom, decreased O2 saturation	True positive
No.14	0	40.6	Positive	0	0	0	Negative	Initial		**N**/N/N	None	False positive
No.15	0	41.0	Positive	0	0	38.23	Inconclusive	Pre-confirmed	42 days	**I**/I/N/Nx	Fever	True positive
No.16	0	41.1	Positive	0	0	0	Negative	Initial		**N**/N	None	False positive
No.17	0	41.1	Positive	38.03	35.62	39.62	Positive	Initial		**P**/N/N(sp)	None	True positive
No.18	0	41.3	Positive	0	0	0	Negative	Pre-confirmed	17 days	**N**	None	True positive
No.19	0	41.3	Positive	0	0	0	Negative	Pre-confirmed	15 days	**N**	None	True positive
No.20	0	41.3	Positive	0	0	0	Negative	Initial		**N**	None	False positive
No.21	0	41.3	Positive	0	0	0	Negative	Initial		**N**/N/N/N/N/N/N	None	False positive
No.22	0	41.4	Positive	0	0	0	Negative	Pre-confirmed	26 days	**N**	None	True positive
No.23	0	41.4	Positive	0	0	0	Negative	Pre-confirmed	about 50 days	**N**	None	True positive
No.24	0	41.5	Positive	0	38.54	0	Inconclusive	Initial		**I**/N	Fever	True positive
No.25	0	41.5	Positive	0	0	0	Negative	Pre-confirmed	48 days	**N**/N	Fever, headache	True positive

No.26	0	41.5	Positive	0	0	0	Negative	Initial		**N**/N/Nx	None	False positive
No.27	0	41.5	Positive	35.65	36.76	34.42	Positive	Pre-confirmed	16 days	**P**/P	None	True positive
No.28	0	41.6	Positive	0	0	0	Negative	Initial		**N**/N	None	False positive
No.29	0	41.7	Positive	0	0	0	Negative	Initial		**N**/N	None	False positive
No.30	0	41.7	Positive	0	0	0	Negative	Initial		**N**/N	Fever with vomiting, URI symptom	False positive
No.31	0	41.7	Positive	36.11	0	33.99	Inconclusive	Pre-confirmed	15 days	**I**/I/N/N	Pneumonia	True positive
No.32	0	41.7	Positive	35.87	37.72	33.93	Positive	Pre-confirmed	47 days	**P**/N/P	URI symptom	True positive
No.33	0	41.8	Positive	0	0	0	Negative	Pre-confirmed	23 days	**N**/N	Fever, URI symptom	True positive
No.34	0	41.8	Positive	37.94	37.89	36.95	Positive	Pre-confirmed	3 months	**P**	None	True positive
No.35	0	41.9	Positive	37.60	0	38.56	Inconclusive	Pre-confirmed	13 days	**I**/P	Fever	True positive
No.36	0	42	Positive	0	0	0	Negative	Pre-confirmed	3 months	**N**/N	Fever	True positive
No.37	0	42.1	Positive	36.71	0	0	Inconclusive	Initial		**I**/N	Sore throat	True positive
No.38	0	42.1	Positive	0	0	0	Negative	Pre-confirmed	27 days	**N**/Px	URI symptom, decreased O2 saturation, general weakness	True positive
No.39	0	42.1	Positive	0	0	0	Negative	Initial		**N**/I/Px	None	True positive †
No.40	0	42.1	Positive	0	0	0	Negative	Initial		**N**/N	None	False positive
No.41	0	42.1	Positive	0	0	0	Negative	Pre-confirmed	214 days	**N**/N/N	None	False positive
No.42	0	42.1	Positive	0	37.92	0	Inconclusive	Pre-confirmed	27 days	**I**/P	General weakness, decreased O2 saturation	True positive
No.43	0	42.2	Positive	0	0	0	Negative	Initial		**N**/I	None	True positive †
No.44	0	42.2	Positive	0	0	0	Negative	Initial		**N**/N	None	False positive
No.45	0	42.4	Positive	0	0	0	Negative	Pre-confirmed	2 months	**N**	None	True positive
No.46	0	42.4	Positive	0	0	0	Negative	Initial		**N**/N	None	False positive
No.47	0	42.6	Positive	0	0	0	Negative	Initial		**N**/N/N/N	None	False positive
No.48	0	42.6	Positive	0	0	38.45	Inconclusive	Pre-confirmed	period unknown	**I**/N	None	True positive
No.49	0	42.7	Positive	0	0	0	Negative	Initial		**N**/N	None	False positive
No.50	0	42.7	Positive	0	0	0	Negative	Initial		**N**/P	None	True positive †
No.51	0	43.0	Positive	36.83	0	0	Inconclusive	Initial		**I**/P/N/N(sp)/I/P	None	True positive
No.52	0	43.4	Positive	0	38.30	0	Inconclusive	Initial		**I**/I	None	True positive

No.53	0	44.1	Positive	0	0	0	Negative	Initial	**N**/N/N/N/P (detection after 18days)	None	False positive
No.54	0	44.4	Positive	0	0	0	Negative	Initial	**N**/N	URI symptom	False positive

Abbreviations: COVID-19, coronavirus disease 2019; E, envelope gene; F/U, follow up; I, inconclusive; N, negative; N2, nucleocapsid gene; o, outside clinic data; P, positive; RdRp/S, RNA-dependent RNA polymerase/S gene encoding spike protein; rRT-PCR, real-time reverse transcription-polymerase chain reaction assay; SARS-CoV-2, severe acute respiratory syndrome coronavirus 2; sp, sputum; x, xpert; Xpert, Xpert Xpress SARS-CoV-2 assay

*COVID-19 results according to rRT-PCR (Allplex or Standard M nCoV Real-Time Detection kit) conducted within one month after the emergency room visit. An x mark after the N or P designation represents data evaluated using Xpert testing; the rRT-PCR data used for comparison is highlighted in bold.

†Early COVID-19 detection using Xpert testing.
